# Whole genome sequence of *Cercospora citrullina* strain UNL090101, the causal agent of watermelon spot disease

**DOI:** 10.1128/mra.00323-25

**Published:** 2025-08-11

**Authors:** Haiyan Wang, Shouchang You, Meng Yang, Jiali Shao, Bingkui Jin

**Affiliations:** 1 Weifang Key Laboratory of Plant Genome and Molecular Breeding, UNELL Biotechnology Co., Ltd., Weifang, Shandong, China; 2 Weifang Guopai Agricultural Technology Co., Ltd., Weifang, Shandong, China; Rochester Institute of Technology, Rochester, New York, USA

**Keywords:** whole genome, *de novo*, *Cercospora citrullina*, watermelon, spot disease

## Abstract

*Cercospora citrullina,* the fungal pathogen responsible for watermelon leaf spot disease, was sequenced through PacBio RS II and DNBSEQ-T7 platforms. A total of 37,403,258 bp was assembled into 19 scaffolds, with scaffolds N50 value of 4,381,129 bp.

## ANNOUNCEMENT

*Cercospora citrullina* primarily infects cucurbit crops, including watermelon (1), melon (2), waxy gourd, and pumpkin (3), causing leaf necrosis. In 2023, this pathogen was first reported in China to infect the seedlings (cotyledons), fruits, and vines of watermelon (4). Given the substantial economic importance of watermelon in agricultural production (FAOSTAT, http://www.fao.org/faostat/en/), conducting whole-genome research on *C. citrullina* is expected to enhance our understanding of plant-fungus interactions, pathogenic mechanisms, and crop resistance.

In this study, *de novo* sequencing of the whole genome was performed on *C. citrullina* strain UNL090101 isolated from a watermelon plant exhibiting clear symptoms of leaf spot disease in Weifang (36.48°N, 119.9°E), Shandong province, China (4). The detection of *C. citrullina* strain UNL090101 was conducted based on concatenated sequences of ITS–ACT–TEF-1–HIS using the maximum likelihood method (4).

Pure cultures were inoculated into potato dextrose medium and incubated at 25°C with shaking for 10 days. Genomic DNA was extracted from the samples using MGIEasy Microbial DNA Extraction Kit (MGI, Shenzhen, China). About 1 µg genomic DNA was randomly fragmented by Covaris. The fragmented genomic DNA was selected by Agencourt AMPure XP-Medium kit (Beckman, USA) to an average size of 200–400 bp for DNBSEQ-T7 platform sequencing library preparation. The double-stranded PCR products were heat denatured and circularized by the splint oligo sequence. The single-strand circle DNA (ssCir DNA) was formatted as the final library. Libraries, qualified by QC, were sequenced: ssCir DNA molecule formed a DNA nanoball (DNB) containing more than 300 copies through rolling-cycle replication. The DNBs were loaded into the patterned nanoarray by using high-density DNA nanochip technology. Finally, pair-end 150 bp reads were obtained by combinatorial Probe-Anchor Synthesis (cPAS) kit. The SMRTbell library (20 kb) for PacBio sequencing was constructed using the SMRTbell Express Prep kit v.2.0 (Pacific Biosciences, Menlo Park, CA, USA). Whole-genome sequencing of *C. citrullina* strain UNL090101 was performed on the PacBio RSII and the DNBSEQ-T7 platforms at Shenzhen Huada Intelligent Manufacturing Technology Co., Ltd. (Shenzhen, China).

The DNBSEQ sequencing results were filtered using SOAPNuke v1.5.6 (5), yielding 17,476,260 total reads and 2,553 Mb(68×) clean data. The PacBio CCS (Circular Consensus Sequencing) data showed 3,068 Mb(82×) data, the CCS number of 187,908, CCS N50 of 16,610 bp, and CCS max length of 44,292 bp. Genome assembly and correction were conducted using Falcon v0.3.0(-v-dal8-t32-h60-e.96-1500-s100-H3000), hifiasm v.0.17.4-r455 (6) and GATK v.1.6-13(-cluster 2-window 5 -stand_call_conf 50-stand_emit_conf 10.0-dcov 200 MQ0 >= 4) (7) for all the reads, and generated 19 scaffolds of 37,403,258 bp. The genome exhibited a GC content of 50.74%, scaffolds N50 value of 4,381,129 bp, N90 value of 2,667,669 bp, max length of 5,909,064 bp min length of 17,535 bp, and with the Gap number of 0 bp. Furthermore, gene composition analysis was conducted using GeneMarkers v.4.21 (8) and Augustus v.3.2.1 (9), predicting a total of 13,025 coding genes, which cover 53.51% of the genome.

Unless otherwise stated, all analyses were performed using default parameters.

## Data Availability

The genome sequence has been deposited in the Sequence Read Archive (SRA) database (Submission number: SRR32486125; BioProject: PRJNA1228159) in NCBI and is publicly accessible.This Whole Genome Shotgun project has been deposited at DDBJ/ENA/GenBank under the accession JBPQGD000000000. The version described in this paper is version JBPQGD010000000.
